# Stramenopile microalgae as “green biofactories” for recombinant protein production

**DOI:** 10.1007/s11274-021-03126-y

**Published:** 2021-08-28

**Authors:** Imke de Grahl, Sigrun Reumann

**Affiliations:** Plant Biochemistry and Infection Biology, Institute of Plant Science and Microbiology, Universität Hamburg, Ohnhorststr. 18, 22609 Hamburg, Germany

**Keywords:** Antimicrobial peptide, Leader sequence, Promoter, Recombinant protein, Subunit vaccine

## Abstract

Photoautotrophic microalgae have become intriguing hosts for recombinant protein production because they offer important advantages of both prokaryotic and eukaryotic expression systems. Advanced molecular tools have recently been established for the biotechnologically relevant group of stramenopile microalgae, particularly for several *Nannochloropsis* species and diatoms. Strategies for the selection of powerful genetic elements and for optimization of protein production have been reported. Much needed high-throughput techniques required for straight-forward identification and selection of the best expression constructs and transformants have become available and are discussed. The first recombinant proteins have already been produced successfully in stramenopile microalgae and include not only several subunit vaccines but also one antimicrobial peptide, a fish growth hormone, and an antibody. These research results offer interesting future applications in aquaculture and as biopharmaceuticals. In this review we highlight recent progress in genetic technology development for recombinant protein production in the most relevant *Nannochloropsis* species and diatoms. Diverse realistic biotechnological applications of these proteins are emphasized that have the potential to establish stramenopile algae as sustainable green factories for an economically competitive production of high-value biomolecules.

## Introduction

Photoautotrophic microalgae are the main primary producers of biomass in the aquatic food chain and play very important roles in marine and freshwater ecosystems. Microalgae naturally synthesize an unprecedented range of valuable bioproducts for human health and animal feed, such as pigments, polysaccharides, antioxidants, and lipids (Borowitzka 2013; Jha et al. 2017). Photoautotrophic cultivation of microalgae is sustainable and cost-efficient, because the organisms directly convert atmospheric CO_2_ to storage carbohydrates without requiring exogenous carbon sources, as do heterotrophic bacteria and fungi. In contrast to sensitive suspension cell cultures of multicellular organisms, upscaling or maintenance procedures are easy and inexpensive for unicellular microalgae (Yang et al. 2016). Initially, applied microalgal research mostly aimed at increasing the lipid content of oleaginous algae for biofuel production, but the production and processing lines were not (yet) cost-competitive with fossil fuels. As an alternative, the high content of health-beneficial polyunsaturated fatty acids and high-value algal products of some microalgae shifted the focus to metabolic engineering strategies and recombinant protein production. Notably, eukaryotic microalgae offer major advantages of traditional prokaryotic and eukaryotic expression systems (Rasala and Mayfield 2015). Similar to prokaryotes, many eukaryotic microalgae grow fast and robustly, and appropriate protocols for their transformation have been reported (Mosey et al. 2021). Unlike bacteria, eukaryotic microalgae are able to correctly modify eukaryotic proteins posttranslationally, for instance, by glycosylation and disulfide bond formation. Both modifications are often required for recombinant proteins to be functional and remain stable in downstream applications (Barolo et al. 2020).

The first genetic tools were primarily developed for the green alga *Chlamydomonas reinhardtii*, which was the first eukaryotic model alga for fundamental and applied research. However, its nuclear transformation often yielded minimal or even negligible levels of recombinant proteins (Schroda 2019). Lately, the large and diverse group of stramenopiles (heterokonts) attracted increasing interest as green biofactories. Stramenopiles are evolutionarily only distantly related to green algae and land plants and evolved by secondary endosymbiosis of a red alga and a heterotrophic protist (Qiu et al. 2013). First sequenced in this group were the diatoms *Thalassiosira pseudonana* and *Phaeodactylum tricornutum* and the Eustigmatophytes *Nannochloropsis gaditana* and *N. oceanica* (Armbrust et al. 2004; Bowler et al. 2008; Radakovits et al. 2012; Vieler et al. 2012). Nowadays, their nuclear genomes can efficiently be transformed and specific antibiotics allow efficient transformant selection (Doron et al. 2016; Mosey et al. 2021). In contrast to *Chlamydomonas*, plastid transformation of stramenopiles remains underdeveloped and unexplored and has only rarely been reported (Xie et al. 2014; Gan et al. 2018).

The present review highlights important recent developments to optimize and expand genetic elements and toolkits for stramenopile algae (*Nannochloropsis* and diatoms) based on nuclear expression towards the realistic ultimate goal to commercially and sustainably produce green bioproducts for diverse intriguing applications. In addition, high-throughput techniques to efficiently evaluate and identify the best expression constructs and transformants are outlined.

## Genetic element choice beyond promoter selection

In order to be economically competitive, recombinant protein production requires high expression rates and protein yields, both of which shall not disturb algal physiology. Moreover, the recombinant protein of interest shall not be reduced in quality and quantity by degradative processes such as proteolysis or autophagy. Suitable expression constructs typically consist of two expression cassettes, namely one for transformant selection conferring antibiotic resistance and one for transgene expression. For high protein production, the promoter is generally the most decisive element. Since the evolutionary distance even among different stramenopile microalgae is large, heterologous transgene expression usually requires the use of endogenous promoters of the same species (Akbari et al. 2014). Hence, one major challenge for each stramenopile host is to identify strong endogenous promoters suitable for stable and efficient production of recombinant proteins from exponential (or linear) to early stationary phase. Well-known constitutive promoters stem from house-keeping genes and cytoskeletal genes (e.g., tubulin), or fundamental processes like translation (elongation factor Tu) and are generally tested first (Radakovits et al. 2012; Vieler et al. 2012). In *N. gaditana* two alternative native promoters, that of heat-shock protein 90 (HSP90) and of the extrinsic protein in photosystem II (EPPSII), increased expression of monomeric Venus (*mVenus*) up to nearly fivefold compared to that of β-tubulin (Ramarajan et al. 2019).

Much pioneering work in stramenopile biotechnology was also conducted for *N. oceanica,* particularly by Eric Poliner from the working groups of Christoph Benning and Eva Farré (Michigan State University, East Lansing, USA). For example, three native bidirectional promoters of *N. oceanica* were recently identified and capable of expressing two transgenes simultaneously and at remarkable intensity (Table 1, Poliner et al. 2020). Best suited for future biotechnological application was the bidirectional promoter controlling gene expression of both nitrate reductase (NR) and the nitrate transporter. In *P. tricornutum*, four constitutive promoters (and terminators) were recently characterized (e.g., the promoter of prohibitin) that possessed a similar expression strength as the widely applied light-responsive promoter of fucoxanthin chlorophyll a/c binding protein B (FcpB, Table 1, Windhagauer et al. 2021). Remarkably and advantageous for recombinant protein production, the new promoters enabled stable reporter gene expression (*mVenus*) under various growth conditions and showed reduced light dependency. Interestingly, this set of promoters was analyzed in a sophisticated transient expression system. A yeast-derived extrachromosomal episome was designed by using a centromere fused with an autonomous replication sequence (CEN/ARS) that prevented plasmid integration into the nuclear system and mediated its replication and segregation to daughter cells (Karas et al. 2015). Meanwhile, a similar episomal expression system has become also available for *N. oceanica* (Table 1, Poliner et al. 2018b).

**Table 1:** Major genetic tools with the potential for high-yield production of recombinant proteins in stramenopile microalgae developed and applied recently

Species	Transformed genome	Genetic element	Information	Reference
*N. gaditana*	Nuclear	Promoter	Endogenous, constitutive promoters of HSP90 and EPPSII	Ramarajan et al. (2019)
			Endogenous, nitrate-inducible NR promoter	Jackson et al. (2018)
*N. oceanica*	Nuclear	Promoter	Endogenous, bidirectional promoters of the NR/NT gene (inducible), of Ribi and of VCP1/2 (both constitutive)	Poliner et al. (2018a, b)
		Leader sequence	14 amino acids of the N-termini of EF and NR	de Grahl et al. (2020), unpublished data
		2A self-cleaving peptide	Optimal P2A length: 60 bp (skipping efficiency > 50%)	Poliner et al. (2017)
		Intron	First intron of VCP1 fused to the corresponding promoter	Südfeld et al. (2021)
		CRISPR-mediated knock-in	Increased HR frequency after targeted integration of DNA double-strand breaks	Naduthodi et al. (2019)
	Plastidic	Promoter	Endogenous, constitutive RbcL promoter	Gan et al. (2018)
	Transient	Extrachromosomal episome	Generation of marker-free mutants by CRISPR/Cas9	Poliner et al. (2018b)
*P. tricornutum*	Nuclear	Promoter	Endogenous, nitrate-inducible NR promoter	Chu et al. (2016)
			Endogenous, constitutive HASP1 promoter	Erdene-Ochir et al. (2019)
			ClP1 promoter of a diatom-infecting virus	Kadono et al. (2015), Pudney et al. (2019)
		Secretion peptide	N-terminal 18 amino acids of HASP1	Erdene-Ochir et al. (2019)
	Plastidic	Promoter	Endogenous, constitutive RbcL promoter	Xie et al. (2014)
	Transient	Extrachromosomal episome, promoter and terminator	Endogenous, constitutive promoters (e.g., of Nub, Pbt, and SVP)	Windhagauer et al. (2021)
*T. pseudonana*	Nuclear	Promoter	Endogenous, silicon-repressible SIT promoter	Davis et al. (2017)

*ClP1* *Chaetoceros lorenzianus*-infecting DNA virus protein, *EF* elongation factor, *EPPSII* extrinsic protein in photosystem II, *HASP1* highly abundant secreted protein 1, *HR* homologous recombination, *HSP90* heat-shock protein 90, *IFT25* intraflagellar transport 25, *NR* nitrate reductase, *NT* nitrate transporter, *Nub* NADH:ubiquinone oxidoreductase, *P2A* 2A peptide of *Porcine Teschovirus*, *Pbt* Prohibitin, *RbcL* RubisCO large subunit, *Ribi* ribosomal subunit bidirectional, *SIT* silicon transporter, *SVP* Synaptobrevin/VAMP-like protein, *VCP* violaxanthin chlorophyll a binding protein

Applications of synthetic and exogenous promoters in stramenopile algae are still in their infancy. Nevertheless, the first promising attempts have already been made. The promoter sources, however, are neither green algae, land plants, nor animals but viruses. In the marine environment, phytoplankton-infecting viruses play a major role in controlling diatom blooms and have a great impact on the composition of marine communities and biogeochemical cycles (Suttle 2007). They often possess strong and specific promoters, as the 35S promoter of the plant pathogen, cauliflower mosaic virus. When analyzing the promoter regions of specific viruses that frequently infect diatoms, several novel conserved motifs were identified (Kadono et al. 2015). These may serve as novel regulatory promoter elements that can possibly be added to expression constructs in future studies to improve recombinant protein production in *P. tricornutum*. Highest *egfp* expression was achieved from the so-called CIP1 promoter of a putative replication-associated protein (VP3) of a DNA virus infecting the diatom *Chaetoceros lorenzianus* (Table 1, Kadono et al. 2015).

Inducible promoters are desirable for the production of recombinant proteins that have a negative impact on algal physiology including toxic proteins. Best characterized and most often applied has been the above-mentioned NR promoter, which is unidirectional in most organisms (Table 1, Chu et al. 2016; Jackson et al. 2018; Poliner et al. 2018a). The NR promoter is induced in the presence of nitrate and repressed by ammonium in many organisms. We recently used these promoter properties from *N. oceanica* to develop an auto-induction system for heterologous gene expression and to fine-tune the kinetics of transgene expression. Thereby, gene expression could be specifically induced at higher cell densities without negatively affecting protein yield and quality (de Grahl et al. 2020). Another inducible promoter is that of the high-affinity silicon transporter (*SIT*) from the diatom *Thalassiosira pseudonana.* Expression of *SIT* was specifically upregulated at ≤ 30 µM silicic acid. Usage of this promoter allowed inducible and efficient expression of a cytotoxic protein during stationary growth upon removal of silicic acid (Table 1, Davis et al. 2017).

Eukaryotic terminator sequences are well-known to increase mRNA stability by polyadenylation, and to raise the translation efficiency, both for endogenous and also heterologous genes. In general, their effect is less pronounced compared to promoters. Commonly used terminator sequences in diatoms and other stramenopiles include those of NR, FcpA/B, β-tubulin, and lipid droplet surface protein (e.g., Niu et al. 2012; Zienkiewicz et al. 2017; Ramarajan et al. 2019).

The incorporation of introns, either placed directly downstream of the promoter or into the transgene CDS, can enhance recombinant protein production upon nuclear expression. This has been demonstrated so far only for *C. reinhardtii*, the genome of which is particularly rich in introns (7.3 introns per gene, Schroda 2019; Baier et al. 2020). In contrast, *N. oceanica* contains only 1.7 introns per gene (Vieler et al. 2012). Hence, translation of the intron effect to other microalgae shall be done with caution and only after experimental effect validation. In *N. oceanica*, the promoter of the violaxanthin chlorophyll a binding protein (VCP1) was extended by the first intron of *VCP1* (Table 1, Südfeld et al. 2021). However, because so far no comparative analyses with the corresponding intron-less promoter are available, future studies shall address whether introns also have an enhancer effect on transgene expression in stramenopile algae.

## Small peptides and their advantages for recombinant protein production

A leader sequence (LS) is a short N-terminal peptide that generally stems from the protein of a strongly expressed gene and often belongs to the chosen promoter. By adding the LS as an N-terminal extension to the recombinant protein, the translation efficiency of transgenes could be further enhanced in many cases. Initially, LS were applied in plastidic expression systems of land plants like tobacco but also proved to work efficiently in *Chlamydomonas* (Kuroda and Maliga 2001; Richter et al. 2018). Recently, we could increase the yield of two recombinant proteins in *N. oceanica* upon nuclear expression by the addition of different LS. A threefold increase of Venus fluorescence was achieved by reporter protein extension with the 14-amino acid LS of NR (de Grahl et al. 2020). Unfortunately, the enhancer effect of one specific LS on translation efficiency often changes with the transgene (Richter et al. 2018), requiring multiple combinatorial analyses of gene element compatibility. Furthermore, so-called *Kozak* sequences, which are short consensus sequences located at eukaryotic initiation sites of translation, can improve the rate of mRNA translation. A novel *Kozak* sequence has been identified for *N. oceanica* and awaits its application in recombinant protein production (Table 1, Dehoff and Soriaga 2014).

Upon nuclear expression, recombinant proteins normally accumulate in the cytosol. If adding an N-terminal signal peptide, recombinant proteins can be secreted across the plasma membrane into the apoplast or the extracellular medium. The later strategy allows direct harvest of recombinant proteins from the medium (e.g., upon microalgae sedimentation by centrifugation) and facilitates further protein purification. This strategy has been well established for *E. coli* and *Pichia pastoris.* As prerequisite, suitable signal peptides need to be identified first. The correct prediction of signal peptides is particularly challenging in stramenopile algae. As a relic of secondary endosymbiosis, stramenopiles possess unusually complex organelle membranes. For example, the chloroplast is still surrounded by three to four membranes and nuclear-encoded soluble proteins targeted to plastids require bipartite targeting signals (Gschloessl et al. 2008). When the secreted proteome of *P. tricornutum* was analysed, one highly abundant protein was identified (Table 1). When its predicted 18-amino acid signal peptide was placed in front of GFP, however, only a minor portion (approx. 10%) of the reporter protein was secreted, requiring further optimization (Erdene-Ochir et al. 2019).

By using short so-called 2A peptides, multiple transgenes present in polycistronic mRNA can be expressed from one single promoter also in microalgae. These short peptides are of viral origin, e.g., from the foot and mouth disease virus. They cause ribosome skipping during translation and separate polypeptide chains. After initial technology application in *Chlamydomonas* (Schroda 2019), method transfer to stramenopile algae was indeed successful. While the above-mentioned viral 2A peptide resulted in only very low ribosome skipping efficiency (< 10%) in *N. oceanica*, the effect caused by the *Porcine teschovirus* peptide (P2A) was > 50% if its length was extended to 60 bp. This peptide subsequently served to metabolically engineer *N. oceanica* and to simultaneously express multiple fatty acid desaturases with the aim to increase the yield of polyunsaturated fatty acids (Poliner et al. 2017). Because the viral origin of the 2A peptide and its length often crucially determine the efficiency of peptide bond skipping, it is recommended to carefully optimize the 2A peptide for each microalgal species.

## CRISPR/Cas9 applications to boost recombinant protein production

Recombinant protein production can possibly be further boosted by advanced genome editing like the CRISPR/Cas9 technology, which has been established by now for most biotechnologically relevant microalgal species (e.g., reviewed by Zhang et al. 2019, Patel et al. 2019). If the introduction of site-specific Cas9-mediated double-strand breaks is combined with homologous recombination, this allows the integration of expression constructs into very specific regions of the nuclear host genome. Those may be regions of very high transcriptional activity to achieve high recombinant protein yields. However, the natural frequency of homologous recombination is usually rare in stramenopiles. To investigate whether a transgene can successfully be integrated into the nuclear genome of *N. oceanica* in a site-specific manner, a double-strand break was first introduced into the *NR* gene by CRISPR/Cas9 using the corresponding guide RNA. Subsequently, the *NR* gene could indeed be replaced by the coding gene of the zeocin resistance marker by homologous recombination, as it was flanked by two 1 kbp-long DNA sequences located up- and downstream of the *NR* gene. By the preceding site-specific double-strand break, the frequency of homologous recombination and the specific gene exchange could be considerably improved (Table 1; Naduthodi et al. 2019).

Taken together, numerous advanced molecular tools have become available for microalgae and offer a great potential to improve recombinant protein production, particularly if several tools are combined and applied in parallel.

## High-throughput strategies for large-scale construct and strain selection

Novel genetic elements and tools need to be carefully evaluated regarding their ability to increase recombinant protein productivity by the host. The expression level of transgenes does not only depend on numerous single cassette elements (see above) but also on their combination compatibility, the success of which is largely unpredictable. For this reason, a large number of different expression cassettes typically need to be compared in microalgae to identify the best (Schroda 2019). Moreover, individual transformants containing the same expression cassette but integrated at different genomic sites often differ significantly in expression strength, referred to as positional effects. This requires analysis and averaging of a large number of transformants for each construct. Therefore, systematic screening strategies are needed particularly in microalgal biotechnology.

To facilitate quantification, recombinant proteins can be extended by fluorescent or luminescent reporters, the protein concentration of which can be easily and accurately determined. The protein of interest is generally extended C-terminally with the reporter. For *N. oceanica* two luciferase variants have been established and their activity can be quantified by chemiluminescence (Poliner et al. 2020). Proteinaceous fluorophores allow subcellular targeting analyses by confocal microscopy and quantification of cellular fluorescence by microplate reader and flow cytometry. Among several possible fluorophores, the monomeric mVenus has been most widely used in *N. gaditana, N. oceanica,* and *P. tricornutum* (e.g., Ramarajan et al. 2019; de Grahl et al. 2020; Windhagauer et al. 2021). The fluorophore is bright, stable, and rather insensitive to bleaching, and its light emisssion does not interfere with chlorophyll autofluorescence (Nagai et al. 2002).

Flow cytometry is a well suitable large-scale screening method to quantify differences in fluorophore expression and concentration in microalgal transformants. By pooling a large number of individual transformants, positional effects on transgene expression can be averaged and the best expression cassette elements identified. Such strategies were, for example, applied in *N. gaditana* and *N. oceanic* (Ramarajan et al. 2019; de Grahl et al. 2020). Fluorescence-activated cell sorters even allow the isolation of single cells showing maximum fluorophore concentrations, even of the tiny *Nannochloropsis* cells (d = 3–5 µm).

Despite their advantages and broad usage, the stable conformation and considerable size of GFP derivatives (27 kDa) and related fluorophores may sterically hinder full functionality of the recombinant protein and often requires tag separation. For tag removal, several strategies are available like the introduction of a proteolytic cleavage site (e.g., for the serine protease thrombin) between the protein of interest and the reporter to cleave both proteins when needed (Charlton and Zachariou 2011). Alternatively, 2A peptides may be inserted between both fusion partners for separation by ribosome skipping in vivo (see above).

## Biopharmaceuticals produced by stramenopiles for aquaculture and human medication

**Fig. 1:**
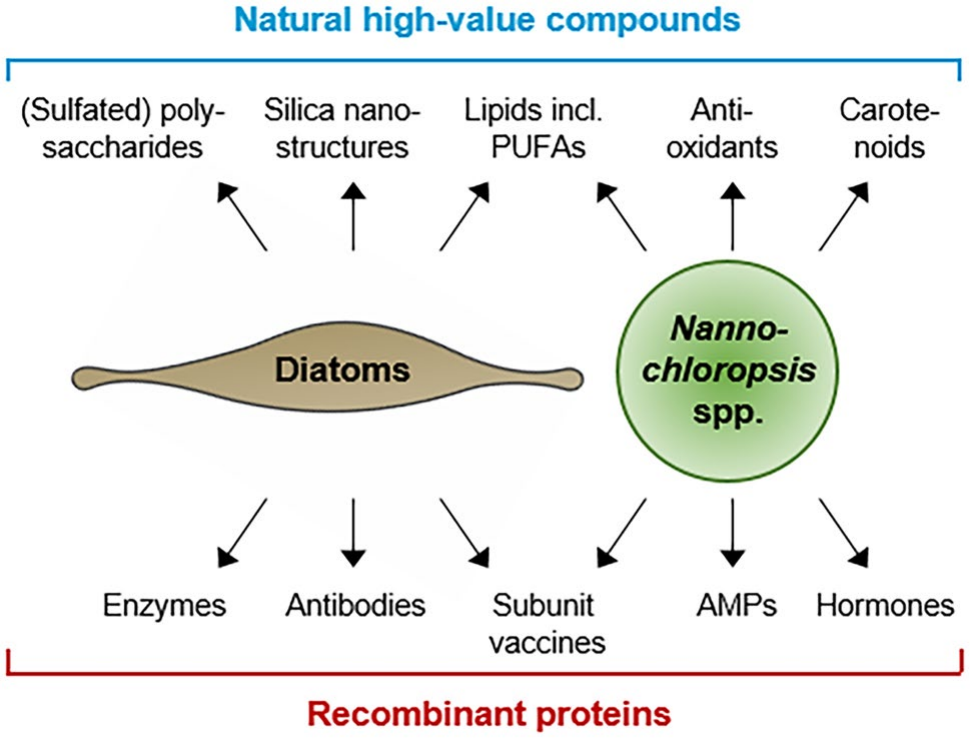
Natural high-value biomolecules and recombinant proteins produced by stramenopile microalgae for biotechnological and biopharmaceutical applications in aquaculture and for human health

Nowadays, aquaculture contributes more than 50% to the worldwide fish demand as human food and animal feed but is prone to lethal bacterial and viral infections (Charoonnart et al. 2018). Microalgae are considered particularly suitable for oral medicine administration of fish since they are already used as fish feed additives to deliver PUFAs and carotenoids. Strategies have been developed to engineer microalgae to produce either vaccines, antimicrobial peptides (AMPs) and even growth hormones (Fig. 1, Shah et al. 2017). For oral administration, the rigid algal cell wall is rather advantageous because active compounds remain long bioencapsulated until digestion in the fish intestines (Criscuolo et al. 2019). In regard to legal and public GMO concerns, the dried dead biomass of microalgae offers major advantages because (i) it does not bear any risk to contaminate human food or the environment, (ii) the material is no longer categorized as GMO, and (iii) its application as feed or food additive is not restricted by GMO laws (Charoonnart et al. 2018; Torres-Tiji et al. 2020).

Beyond aquaculture, microalgae are also used for the production of recombinant proteins for human medication (Fig. 1). For these applications, the capability of eukaryotic microalgae to perform posttranslational modifications like disulfide bond formation and glycosylation upon nuclear expression is prerequisite and often crucial for native protein functionality. Many microalgae have been classified as “Generally Recognized As Safe” (GRAS) for human consumption. Together with the above-mentioned non-GMO status of dead algae, this allows direct oral administration of whole algal cells (Criscuolo et al. 2019). Although stramenopiles like *Nannochloropsis* are not yet included in this classification, the overall chemical composition (particularly their content of polyunsaturated fatty acids) and the absence of toxins are important features for these microalgae to be introduced to human nutrition and to be used as vehicles for medicine delivery in the near future (Zanella and Vianello 2020).

Subunit vaccines are typically viral or bacterial surface proteins containing antigenic, surface-exposed epitopes. Contrary to injectable vaccines, edible vaccine formulations can trigger superior mucosal immune responses in vertebrates including T-cell mediated immunity. Few subunit vaccines have already been produced successfully in microalgae and indeed mounted specific immune responses in fish (Embregts and Forlenza 2016; Charoonnart et al. 2018). When mice were fed with entire or sonicated cells of *T. pseudonana* that synthesized a protective antigen against the bacterial pathogen *Histophilus somni*, a specific immune response was indeed induced (Davis et al. 2017). We recently engineered *N. oceanica* to produce the surface protein VP2 of the infectious pancreatic necrosis virus, which causes high mortality rates in young salmonids. A high protein yield could indeed be achieved (unpublished data) making *Nannochloropsis* a promising alga for oral vaccine delivery in aquaculture.

Recently, marine antimicrobial peptides (AMPs) became attractive as potential fish feed additives and as novel medication against intestinal fish pathogens to strengthen fish immunity in aquaculture and to solve the global problem of increasing bacterial resistances (Charoonnart et al. 2018). These peptides of approx. 10 to 50 amino acid residues in length are synthesized by eukaryotes by their innate immune system. They possess potent broad-range bactericidal activities. Functionally, most AMPs target microbial cell membranes and cause quick pathogen lysis and death (Zhang and Gallo 2016). Therefore, many AMPs seem to be less susceptible to the development of resistances and are denominated “next generation antibiotics” (Spohn et al. 2019). Several potent AMPs for application in aquaculture have already been produced in *Chlamydomonas* (Shi et al. 2021). Bovine lactoferricin represents the first AMP synthesized by engineered *Nannochloropsis*. Lactoferricin is a 3.1-kDa peptide (24 amino acids) cleaved in vivo from the N-terminal domain of the glycoprotein lactoferrin. When using an exogenous heat inducible promoter from *C. reinhardtii*, the AMP was produced as a dsRed fusion protein at considerable yield (4.3% of total soluble protein) in *N. oculata.* After oral administration of *Nannochloropsis*, Medaka (*Oryzias latipes*) was efficiently immunized, as they showed a 17-fold higher survival rate after infection with *Vibrio parahaemolyticus* (Li and Tsai 2009). Interestingly, the same expression system was applied to produce a fish growth hormone in *N. oculata* (4.2 µg/l culture) that indeed stimulated the growth of red tilapia larvae (*Oreochromis niloticus* × *O. mossambicus*, Chen et al. 2008).

Further biotechnologically-relevant bioactive proteins have also been produced in the diatom *P. tricornutum*. The CIP1 promoter of the above-mentioned virus infecting the diatom *Chaetoceros lorenzianus* was successfully applied to produce a recombinant phytase. Phytases are specific phosphatases that catalyze the hydrolysis of phytic acid (myo-inositol hexakisphosphate), which is an indigestible, organic form of phosphorus found in plant tissue. The enzymes are biotechnologically attractive to improve phosphorus bioavailability of the algal biomass in animal feed. When produced in *P. tricornutum*, a phytase activity of 40,000 units per gram of total soluble protein was achieved (Pudney et al. 2019). Another example is the production of an antigenic Hepatitis B surface protein and the corresponding human monoclonal antibody in *P. tricornutum,* both of which were expressed from the nitrate inducible NR promoter (Hempel et al. 2011). In a follow-up study, the antibody was secreted with high efficiency into the growth medium after the removal of its endoplasmic reticulum retention signal (Hempel and Maier 2012). In both cases, the antibody was fully assembled (two heavy and two light chains) and indeed able to bind to the antigen in vitro with high affinity. Recently, the successful binding of the same algae-made antibody to two different receptors of human immune cells was demonstrated in vivo (Vanier et al. 2018).

## Conclusions

Several recombinant proteins and peptides have already been produced successfully in photoautotrophic stramenopile microalgae, and their hypothesized bioactivities have been proven for the first target organisms. While initial method development focused primarily on *Chlamydomonas*, the transfer of these tools to the more robust and biotechnologically more promising stramenopiles has been intensively pushed forwards by several research groups. Numerous new genetic tools have been developed for nuclear transgene expression in stramenopiles, primarily for diatoms (*Phaeodactylum*, *Thalassiosira*) and *Nannochloropsis*. Well suitable fluorescent and luminescent reporters have been established and optimized for these hosts. Powerful and sophisticated genetic elements have been deciphered and their positive enhancer effects on recombinant protein production have been demonstrated. High-throughput strategies like flow cytometry have become available and will further accelerate identification of the best genetic elements in the most promising hosts. In the near future, the successful production of high-value biopharmaceuticals in *Nannochloropsis* and diatoms is realistic, even at industrial scale and for commercial applications, and will further boost the establishment of the organisms as sustainable green cell factories.

## Data Availability

The authors will make scientific material available upon request.
